# The relationship between executive dysfunctions and quality of life of children and youth with psychiatric disorders

**DOI:** 10.1192/j.eurpsy.2021.603

**Published:** 2021-08-13

**Authors:** B. Engel Yeger, G. Hamud, A. Avital

**Affiliations:** Occupational Therapy, University of Haifa, Haifa, Israel

**Keywords:** quality of life, daily life activities, children and youth, Executive functions

## Abstract

**Introduction:**

The high cognitive abilities named executive functions (EF) are responsible for emotional regulation and for goal-oriented behavior. EF are frequently disrupted in anxiety disorders and negatively affect daily function and quality of life (QoL). Nevertheless, EF evaluation is usually performed in the laboratory using neuro-psychological assessments that refer to specific components (such as working memory, inhibition), but lacks a comprehensive profile of EF and the expressions in real life context.

**Objectives:**

To elaborate the knowledge about EF in daily life of children/youth with psychiatric disorders, by comparing their EF to those of healthy controls, using an ecological measure that imitates daily life scenarios; To examine the relationship between EF and QoL in the study group.

**Methods:**

Participants were 49 children and youth aged 8-18 years: 25 subjects with psychiatric (mainly anxiety) disorders and 24 healthy controls. The children’s parents completed a socio-demographic questionnaire, the Child Behavior Checklist (CBCL) to profile emotional difficulties; The Behavior Rating Inventory of Executive Functions (BRIEF) which examines EF components related to meta-cognition and behavioral regulation; and the Pediatric Quality of Life Inventory (Peds-QoL).

**Results:**

The study group had more EF difficulties [reduced behavioral regulation (F=31.81; p<.001) and metacognition (F=26.25; p<.001)], and lower QoL. In the study group, EF difficulties correlated with reduced physical, emotional, social, and school-related QoL.

**Conclusions:**

EF should be evaluated in children/youth with psychiatric disorders, by ecological evaluation that reflect the difficulties in daily life. This may focus intervention on child’s specific needs and improve the outcomes in terms of better function, development and QoL.

